# Effect of Mobile Health Technology on Weight Control in Adolescents and Preteens: A Systematic Review and Meta-Analysis

**DOI:** 10.3389/fpubh.2021.708321

**Published:** 2021-07-15

**Authors:** Jui-Mei Yien, Hsiu-Hung Wang, Ruey-Hsia Wang, Fan-Hao Chou, Kuo-Hsiung Chen, Fu-Sheng Tsai

**Affiliations:** ^1^College of Nursing, Kaohsiung Medical University, Kaohsiung, Taiwan; ^2^Department of Health Care Management, University of Kang Ning, Tainan City, Taiwan; ^3^Department of Medical Research, Kaohsiung Medical University Hospital, Kaohsiung, Taiwan; ^4^Department of Business Administration, Cheng Shiu University, Kaohsiung, Taiwan; ^5^Center for Environmental Toxin and Emerging-Contaminant Research, Cheng Shiu University, Kaohsiung, Taiwan; ^6^Super Micro Mass Research and Technology Center, Cheng Shiu University, Kaohsiung, Taiwan

**Keywords:** childhood obesity, overweight, adiposity, digital device, adolescents, preteens

## Abstract

Childhood obesity is a crucial public health concern. In recent years, numerous studies have employed mobile health technology applications for weight control in children but obtaining varying effects. We conducted a meta-analysis to discuss the effectiveness of mobile health technology in reducing the body mass index (BMI) of obese children. The standardized mean difference (SMD) in BMI between the intervention and control groups was employed to measure the effect of mobile health technology intervention on weight control. The Comprehensive Meta-Analysis Version 3 software was employed for meta-analysis, and the results are presented in a forest plot. This study included nine randomized control trials, which featured a total of 1,202 participants. The meta-analysis revealed that mobile health technology intervention did not have a significant weight loss effect on subjects with obesity. However, by using ethnicity as a moderating variable for subgroup analysis, we discovered that the BMI of ethnic Chinese groups who received mobile health technology intervention was significantly lower than that of the control group. This effect was not significant in the non-ethnic Chinese subgroup. Therefore, the use of mobile health technology intervention for weight control in ethnic Chinese children resulted in significantly lower BMI in these children; however, the use of mobile health technology intervention for weight control is unsuitable for non-ethnic Chinese children.

## Introduction

Childhood obesity is a crucial, global health problem that has raised concerns among public health experts. However, the prevalence rate of childhood obesity in recent years has remained unchanged (1). Different from traditional weight loss intervention, which involves face-to-face health education, scholars have endeavored to identify alternate methods to provide enhanced weight loss effects (2, 3). Children and adolescents frequently use mobile health technologies to retrieve information from the Internet and interact with online communities in their daily lives; therefore, mobile health technologies are a feasible channel for providing health information (4). Recently, mobile health technologies have been applied to manage children's weight and prevent obesity (5). Some scholars have done similar studies before, but the included literature includes non-randomized trials (6). Randomized controlled trials (RCTs) are the inclusion criteria for this study. The database search date is also the most recent; therefore, there is newer discovery in our research.

Due to limited time and resources, health experts cannot timely monitor and support patients in their everyday lives. This limitation can be overcome by the development of self-health management technologies (6). Because technology is commonly used in the daily lives of most people, the use of mobile health technologies for weight control presents advantages of high accessibility and adherence (7). In theory, the use of mobile health technologies for weight control in children is effective; after intervention, real-time feedback can be obtained, which maintains the motivation for weight loss in users (8). Mobile health technology is an acceptable and feasible method for reducing childhood obesity. However, large heterogeneity in research design were found across different studies (9, 10). Therefore, a consensus regarding the efficiency of mobile health technology applications in weight control in children has yet to be reached (11). This may or may not be true. However, it is true that despite the contentions there are many studies demonstrating the effectiveness of these technologies. In fact, some groups have cautiously supported their use as an adjunct to weight management interventions. Therefore, the objective of this study was to examine the effect of mobile health technology on weight control in adolescents and preteens.

## Methods

### Search Strategy and Inclusion Criteria

A meta-analysis was conducted to evaluate the weight control effect of mobile health technology on subjects with obesity. The researchers searched the Embase, Medline, Cochrane Library, Web of Science, and ScienceDirect databases for articles on mobile health technology interventions among subjects with obesity. The literature collection period ended in February, 2021; the search methods involved using suitable controlled vocabulary and free-text terms. The keywords used for searching included childhood obesity, pediatric obesity, electronic technology, smartphone, activity tracker, mobile device, mobile application, and fitness tracker. The search syntaxes used are detailed in the Appendix. Inclusion criteria include RCTs or comparative experimental research. All searched studies comprised at least two participant groups, namely the intervention group and control group. The target population consisted of children and adolescents with obesity risks. In the included literature, intervention was conducted during the follow-up period by using smartphones, social medial, or follow-up instruments.

The selected studies (including RCTs and review articles) were manually screened to determine their inclusion or exclusion in the final analysis. In the first phase, article titles and abstracts were reviewed, and single-arm studies, case series, and case reports were excluded. In the second phase, the researchers reviewed the entire articles, and exclusion criteria included the following: non-RCTs and studies with participants who were older than 18 years of age, absence of BMI data, studies containing participants with physical disability, and interventions not involving mobile health technology. In addition, childhood obesity caused by drug use or diseases was not included in the scope of this study.

### Data Extraction and Quality Assessment

After the selected articles underwent a review by the two reviewers, the researchers used a predetermined format to extract article data, primarily the first authors, study periods, sample sizes, participant characteristics, and intervention contents. The two reviewers independently used the Jadad scale to evaluate the quality of the RCTs (12). Jadad scoring is generated across three dimensions, namely randomization (2 points), blinding (2 points), and withdrawals and dropouts (1 point). The total score ranges from 0 to 5; higher scores indicate higher literature quality (12, 13). If the reviewers' opinions differed, a discussion was held between the reviewers under the coordination of a corresponding author until a consensus was reached.

### Data Integration and Analysis

BMI is the most commonly used obesity evaluation index and is used by the World Health Organization (WHO) (14), rendering it suitable for meta-analysis; it consists of simple calculations and easily accessible data (15). BMI mean and standard deviation are viable for statistical analysis. Therefore, we used BMI to evaluate weight loss effects. In particular, we used the postintervention standardized mean differences (SMDs) between the intervention and control groups to evaluate the intervention effect. The 95% confidence interval (CI) of the overall SMD was negative, indicating that the weight loss effect in the intervention group was greater than that in the control group. The Comprehensive Meta-Analysis Version 3 (Biostat, Englewood, NJ, USA) was used for analysis. We used *I*^2^ to assess the degree of heterogeneity among the studies. When *I*^2^ > 50%, the included studies had high heterogeneity (16). The random-effect model was used to compile the SMDs in all selected studies and to further identity variables for subgroup analysis. In addition, Egger's test was used to determine potential publication bias. Statistical significance was set as *p* < 0.05 (17); if p was not significant, the number of the included studies was sufficient for meta-analysis.

## Results

### Literature Search and Characteristics of Included Studies

**Figure 1:**
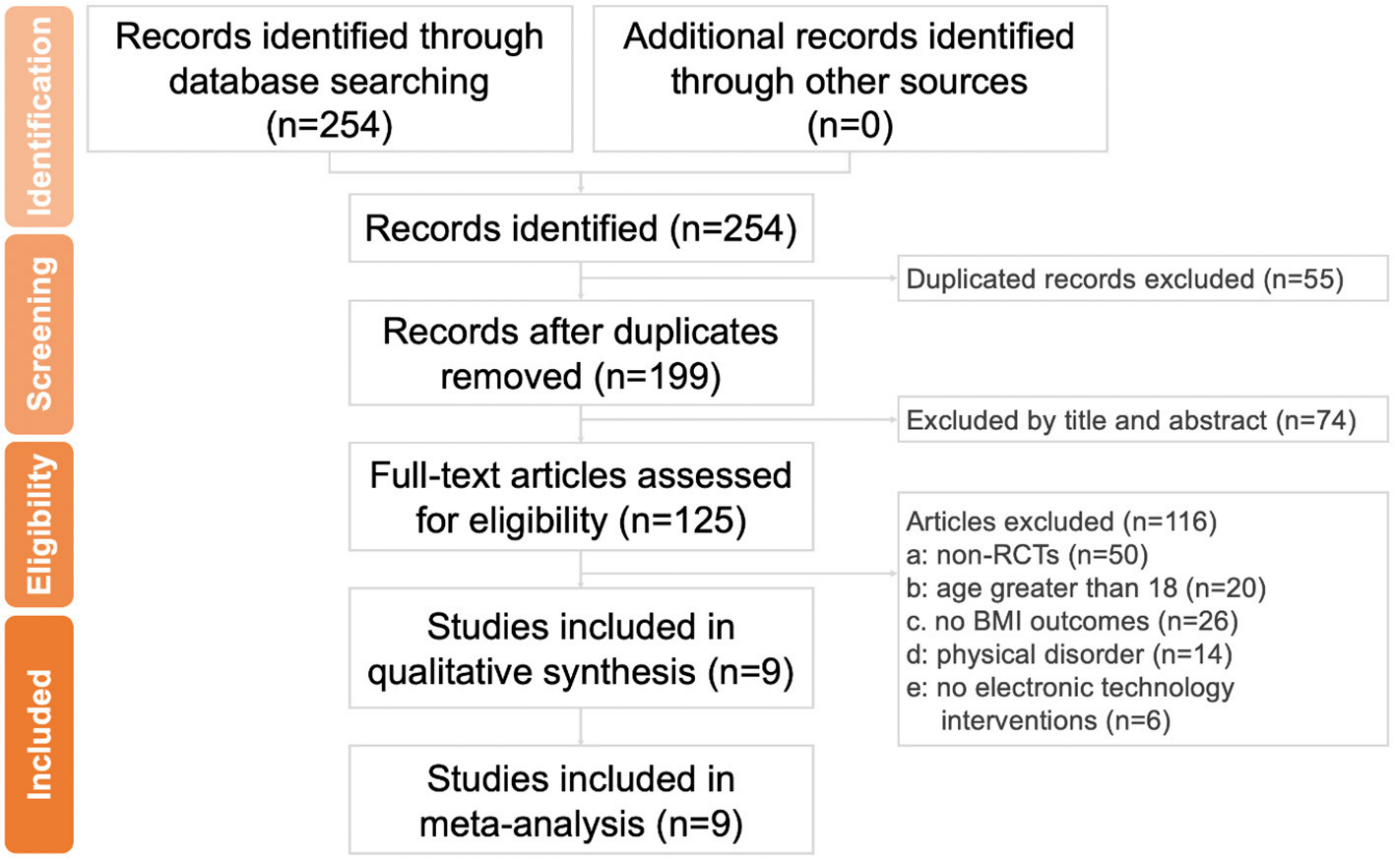
Flow diagram of the study selection process.

The article selection was conducted in accordance with the Preferred Reporting Items for Systematic Reviews and Meta-Analysis (PRISMA) (Figure 1). A total of 254 citations (97, 83, and 74 from Embase, Medline, and Cochrane Library, respectively) were collected. After exclusion of 55 repeated citations, 199 remained. After a review of the titles and abstracts, 74 unsuitable citations were eliminated. Finally, the researchers reviewed the full text of 125 studies. By applying the exclusion criteria, the researchers excluded 50 non-RCT studies, 20 studies with participants over 18 years old, 26 studies that did not present their results in terms of BMI, 14 studies in which participants with physical disorders were involved, and 6 studies that did not use mobile health technology inventions. Finally, only 9 double-arm RCT studies were retained for meta-analysis. Regarding participant ethnicity, 3 studies conducted experiments on ethnic Chinese participants from the United States or Hong Kong (18–20) based on sample descriptions of studies; 6 studies were based on non-ethnic Chinese participants from the United States (21, 22), Australia (23–25), and Italy (26).

Finally, 1,202 participants from the included 9 studies were included in the quantified analysis. Overall, the participant age ranged from 8 to 18 years; it ranged from 8 to 18 years for ethnic Chinese participants and 9 to 17 years for non-ethnic Chinese participants. The mobile health technology most often used by the participants included smartphones, wristbands, social media, and short message services, through which participants could engage in weight loss activities and report data. Table 1 lists the characteristics of the participants, the research method used in each study, and the Jadad scores of the included studies. The table reveals that the Jadad scores of the included studies were all >4, indicating favorable research quality.

**Table 1:** Characteristics of the studies included.

**First author, year**	**Country**	**Ethnicity**	**Age**	***N* INT/CTR**	**Intervention (duration)**	**Study duration (months)**	**Jadad score**
Chen et al. (18)	USA	Ethnic Chinese	13–18	23/17	• Wristband: healthy lifestyle behavior tracking (6 months) • Smartphone and computer: online educational program (3-month intervention phase) • Mobile phone: text messages (3-month maintenance phase)	6	5
Chen et al. (19)	USA	Ethnic Chinese	13–18	21/15	• Wearable sensor: healthy lifestyle behavior tracking (6 months) • Smartphone and computer: online educational modules (3 months) • Mobile phone: text messages (3 months)	6	4
Lee et al. (20)	Hong Kong	Ethnic Chinese	8–16	63/52	• Facebook, apps, email, and phone call: social support (6 months)	6	5
Lubans et al. (21)	Australia	Non-ethnic Chinese	12–14	141/153	• Pedometer: self-monitoring of physical activity (12 months) • Text message: social support (12 months)	12	5
Lubans et al. (22)	Australia	Non-ethnic Chinese	12–14	121/143	• Pedometer: physical activity self-monitoring (17 weeks) • Smartphone apps: screen-time reduction (15 weeks)	18	5
Mameli et al. (23)	Italy	Nonethnic Chinese	10–17	16/14	• Wristband: low energy expenditure (3 months) • Smartphone apps: high energy intake (3 months)	3	4
Nguyeen et al. (24)	Australia	Non-ethnic Chinese	13–16	43/50	• Landline telephone: telephonic coaching (24 months) • Mobile phone: telephonic coaching, short message service and/or email communication (24 months)	24	4
Nollen et al. (25)	USA	Non-ethnic Chinese	9–14	19/18	• Smartphone and tablet: real-time goal setting, self-monitoring, tips, and feedback (3 months)	3	4
Smith et al. (26)	USA	Non-ethnic Chinese	12–14	139/154	• Smartphone apps and website: physical activity monitoring, result recording, tailored messaging, peer assessment, and goal setting (15 weeks) • Pedometer: self-monitoring and goal setting (17 weeks)	8	5

*N, Number of participants; INT, Intervention; CTR, Control*.

### SMDs for Weight Control

**Figure 2:**
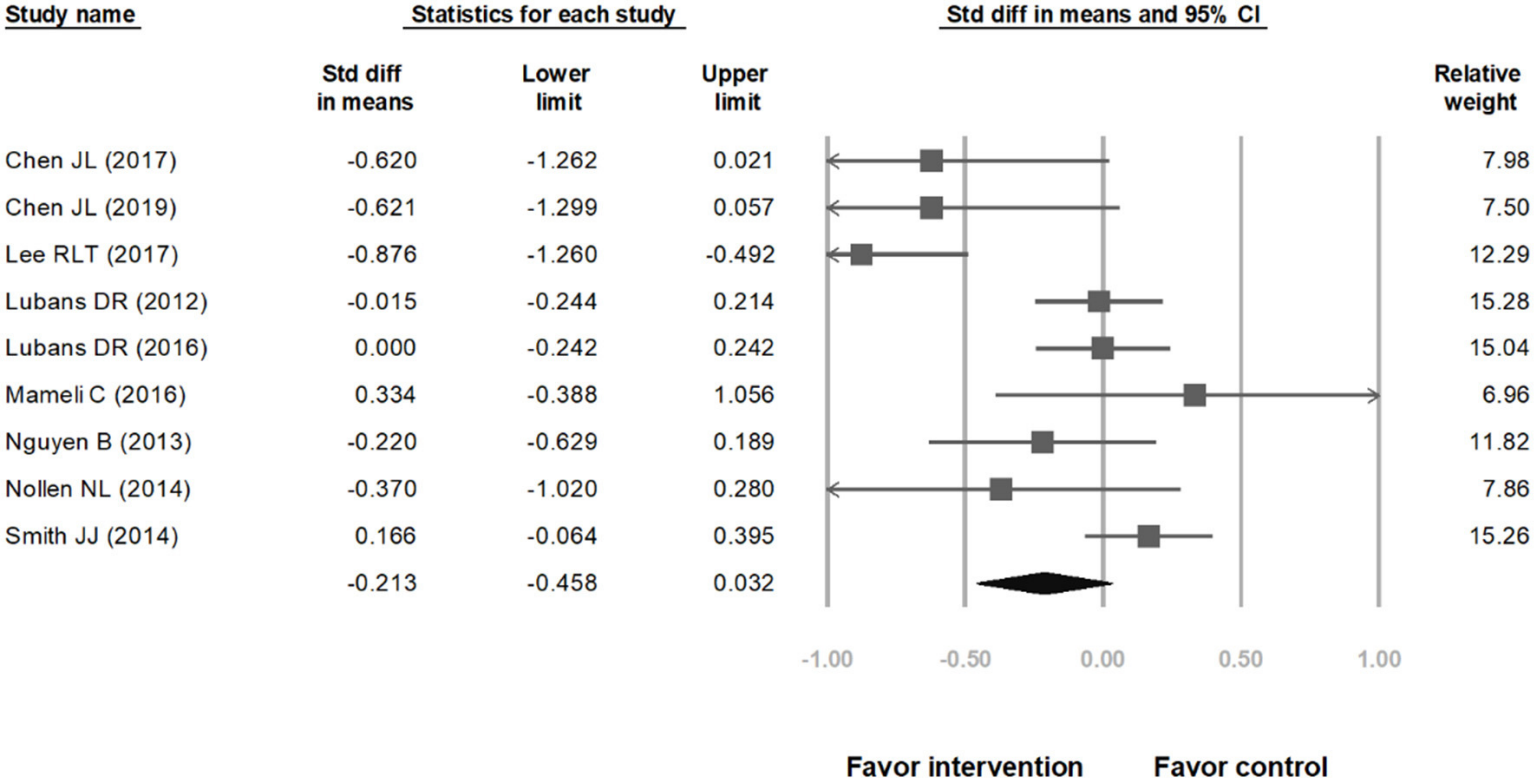
Forest plot showing the effect of mobile health technology on weight control in adolescents and preteens as compared with control groups, overall effect.

**Figure 3:**
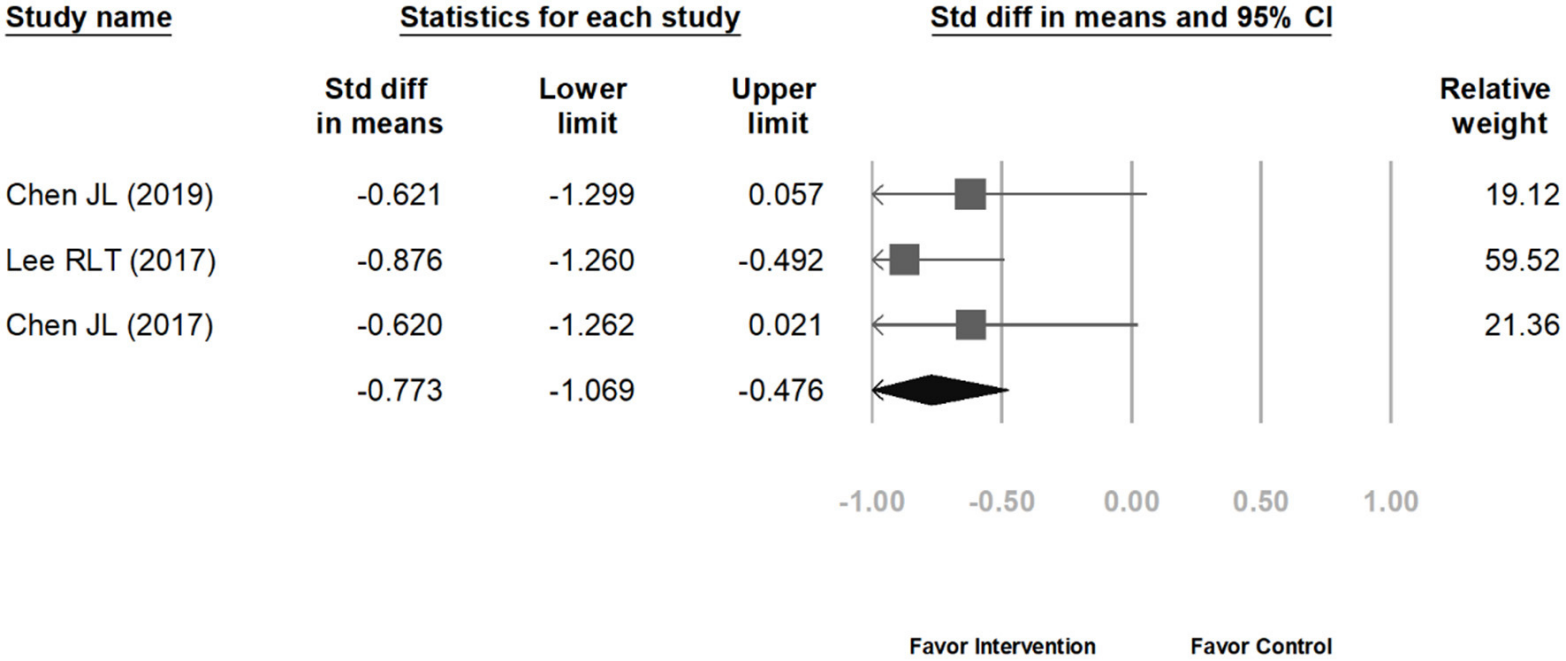
The weight loss effect between the intervention and control groups of the ethnic Chinese participants.

**Figure 4:**
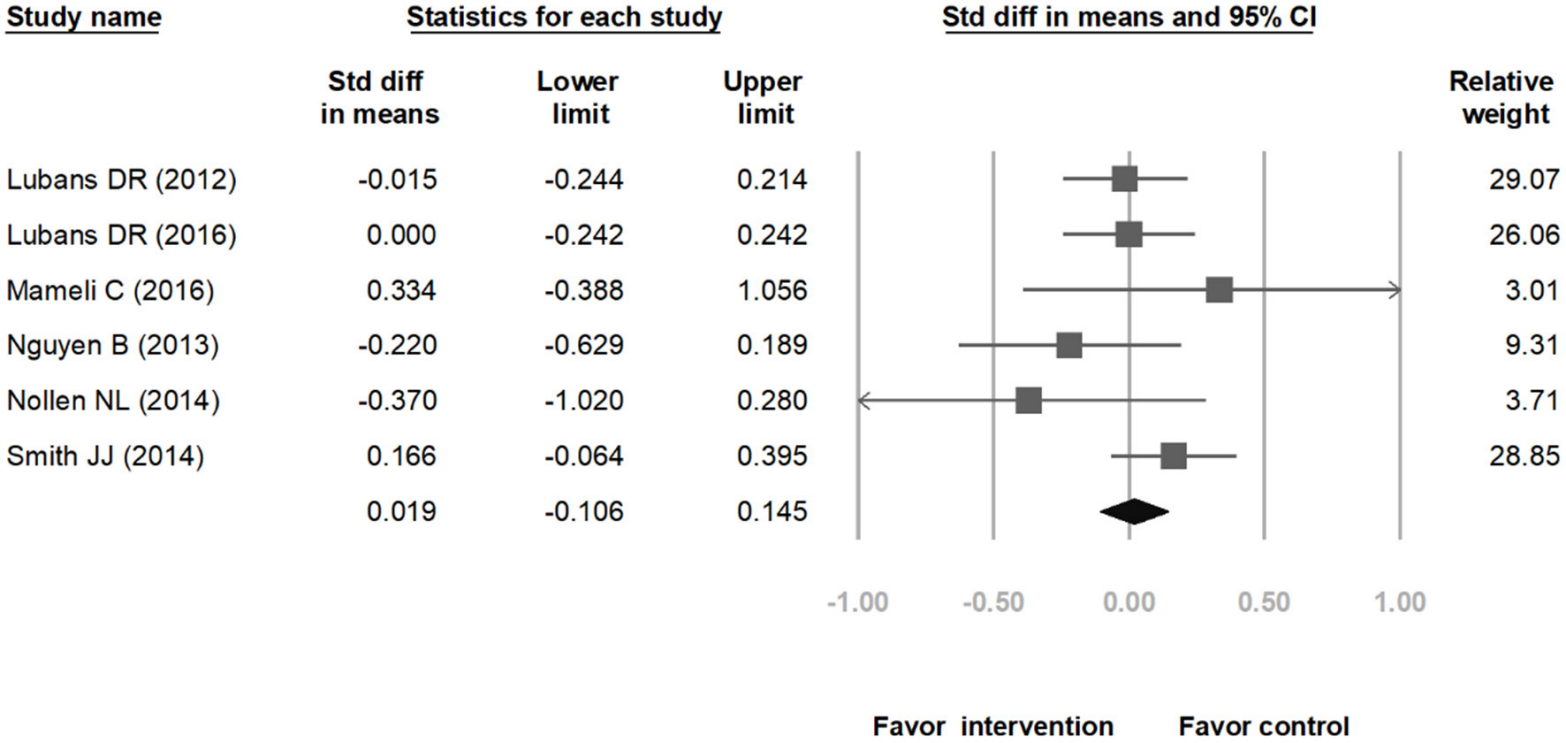
The weight loss effect between the intervention and control groups of the non-ethnic Chinese participants.

The meta-analysis included nine studies on weight loss in children. Figure 2 indicates that the range of 95% CI is shorter and the weighted values are greater for studies with larger sample size. In Figure 2, the overall SMD is −0.213 (95% CI: −0.458 to 0.032). The lower and upper limits of the 95% CI of the overall SMD crossed the vertical center line (SMD = 0.00), indicating that the differences between the intervention groups and their corresponding control groups were non-significant. Subsequently, we computed the overall heterogeneity of the 9 included studies; 25, 50, and 75% heterogeneity represented low, moderate, and high heterogeneity, respectively (27). Because *I*^2^ = 72.56%, the studies exhibited high heterogeneity; therefore, identifying moderating variables for subgroup analysis is necessary. After carefully reviewing these nine studies, there seemed to be a discrepancy in effect on BMI between ethnic Chinese and non-ethnic Chinese subgroups. Therefore, we computed the heterogeneity levels between ethnic Chinese and non-ethnic Chinese participant groups and obtained results of *I*^2^ <0.01% and *I*^2^ = 1.73%, respectively. This indicates that both subgroups exhibited low heterogeneity, thereby verifying that ethnicity is a suitable variable for subgroup analysis. The analysis results in Figure 3 show that the 95% CI of the overall SMD in the ethnic Chinese subgroup was situated to the left of the center line and did not cross it. This indicates that in the ethnic Chinese subgroup, the decrease in BMI in the intervention groups was significantly higher than that in the control groups (SMD: −0.773; 95% CI: −1.069 to −0.476). In addition, Figure 4 indicates that the weight loss effect was not significantly different between the intervention and control groups of the non-ethnic Chinese participants (SMD: 0.019; 95% CI: −0.106 to 0.145). Regarding the publication bias of the overall SMD, the results of Egger's test did not exhibit significance (*p* = 0.146), thereby indicating that publication bias was not present and that the number of included studies was sufficient for the meta-analysis.

## Discussion

This study conducted a meta-analysis to determine the effect of mobile health technology application intervention on weight loss in children. The meta-analysis included 3 and 6 studies on experiments conducted with ethnic Chinese and non-ethnic Chinese participants, respectively. The meta-analysis results indicated that mobile health technology intervention did not have a significant effect on weight loss in children. However, when ethnicity was set as the variable for subgroup analysis, the BMI value in the ethnic Chinese intervention group was lower than that in the ethnic Chinese control group, thereby indicating a significantly greater weight loss effect in the ethnic Chinese intervention group. However, for non-ethnic Chinese participants, the intervention effect was not significant. This indicates that the weight loss intervention may consist of underlying mechanisms unrelated to mobile health technology intervention. We speculate that the intervention effects differed between ethnic Chinese subgroup and non-ethnic Chinese subgroup may be due to cultural factors, psychological factors, and the studies on ethnic Chinese participants having shorter follow-up periods than that of studies on non-Chinese participants.

From a cultural factor perspective, a possible mechanism that influences the intervention effect among ethnicity cultures is differences in body image and aesthetic perception (28). Differences in ethnicity culture result in different perceptions toward different body shapes. This factor may result in different obesity prevalence rates among different ethnicities (29). Ethnic Asian students in the United States exhibited high dissatisfaction toward their body image (30). Ethnic Chinese and non-ethnic Chinese adolescents had different psychological feelings toward body weight. Ethnic Chinese adolescents tended to be dissatisfied with their body weights and feel depressed; this phenomenon was not present in Hispanic adolescents (31). This may be the reason underlying the greater weight loss effect of mobile health technology intervention on ethnic Chinese children.

Unhealthy lifestyle is the main cause of obesity. The loss of motivation to live a healthy lifestyle results in an unbalanced diet and sedentary behavior among individuals (32). Maintaining a healthy body weight requires continuous effort; mobile health technology intervention is suitable for enhancing adolescents' motivation to maintain a regular diet and exercise habits (30). This indicates that in addition to the varying acceptance levels of different ethnicity groups toward mobile health technology intervention, differences in psychological factors among different ethnicities may result in varying weight loss effects (33, 34).

One of the important aspects of these technologies is not only that they are based on the evidence and that they support self-monitoring and goal setting, but that they also provide a sufficient amount or dose of “change talk”—that is motivational cognitive challenges that assist the adolescent in making changes in their behavioral choices and weight related decisions with the knowledge and experience that they inherently have and accrue through the intervention.

In addition, differences in the follow-up period in each study may have resulted in the significantly different temporal effects of weight loss (35). The follow-up period of the 3 studies conducted on ethnic Chinese participants was 6 months, whereas among the 6 studies conducted on non-ethnic Chinese participants, 4 studies consisted of follow-up periods of 8, 12, 18, and 24 months. Therefore, the weight loss effect of mobile health technology intervention on ethnic Chinese participants was only for a short term. Future studies on ethnic Chinese participants with longer follow-up periods are necessary. By using follow-up periods with the same duration, researchers can attain a greater understanding of the different effects of mobile health technology intervention on different ethnicity subgroups.

Based on the study results, we provide suggestions for future research. First, the meta-analysis of this study used mean BMI to measure weight loss results. Future studies may include other obesity measurement indices, such as BMI changes, zBMI, BMI%, body fat, and waist-hip ratio. Next, the meta-analysis only included three studies involving ethnic Chinese participants. Future studies should include more studies on obesity in ethnic Chinese children. Finally, future researchers may conduct long-term follow-up on ethnic Chinese participants to fulfill the current research gap of longitudinal research.

## Conclusions

Mobile health technology applications have been widely used in preventing childhood obesity. However, the results were not as expected. The research results indicate that by using ethnicity as a moderating variable and under the effect of cultural and psychological factors, the use of mobile health technology intervention may reduce obesity in ethnic Chinese children. However, the weight loss effect of mobile health technology intervention in other ethnicities remains uncertain. Therefore, ethnicity culture must be used as a key consideration in future studies using mobile health technology interventions to reduce childhood obesity.

## Data Availability Statement

The raw data supporting the conclusions of this article will be made available by the authors, without undue reservation.

## Author Contributions

R-HW, H-HW, and F-HC: conceptualization, methodology, and validation. J-MY: data curation, formal analysis, and writing original draft. J-MY and K-HC: investigation. R-HW: project administration, resources, and supervision. H-HW and F-HC: resources. K-HC: software. F-ST: visualization, writing-review, and editing.

## Conflict of Interest

The authors declare that the research was conducted in the absence of any commercial or financial relationships that could be construed as a potential conflict of interest.

## Figures and Tables

**Table:** 

**#**	**Search syntax**
1	(((childhood OR pediatric OR child* OR pupil OR adolescent OR teenage* OR juvenile OR kid) NEAR/4 (obesity OR overweight OR adiposity OR “weight management” OR “weight control” OR “weight loss” OR weight-loss)) OR ((obese OR overweight) AND (child* OR kid OR pupil OR adolescent))):ti,ab,kw,de
2	“childhood obesity”/exp
3	((smartwatch OR wristband OR mHealth OR “mobile health” OR “activity tracker” OR “fitness tracker”) OR ((mobile OR electronic OR digital OR cloud OR wearable OR smart OR health OR fitness OR wrist-worn OR biofeedback OR wireless OR internet) NEAR/4 (tracker OR sensor OR application OR app OR device OR technolog* OR watch OR product OR wearable))):ti,ab,kw,de
4	“activity tracker”/exp OR “mobile health application”/exp OR “self-monitoring”/exp
5	(BMI OR “body mass index”):ti,ab,kw,de
6	“body mass”/exp
7	(#1 OR #2) AND (#3 OR #4) AND (#5 OR #6) AND [embase]/lim
8	#7 AND (“crossover procedure”:de OR “double-blind procedure”:de OR “randomized controlled trial”:de OR “single-blind procedure”:de OR (random* OR factorial* OR crossover* OR cross NEXT/1 over* OR placebo* OR doubl* NEAR/1 blind* OR singl* NEAR/1 blind* OR assign* OR allocat* OR volunteer*):de,ab,ti)

## Appendix

**Embase**
